# Genetic risk factors for respiratory diseases of preterm infants

**DOI:** 10.1186/2194-7791-1-S1-A3

**Published:** 2014-09-11

**Authors:** Friederike Pagel, Sarah Leick, Michael Preuß, Christoph Härtel, Egbert Herting, Wolfgang Göpel

**Affiliations:** Department of Paediatrics, University of Lübeck, UKSH, 23538 Lübeck, Germany; Institute of Medical Biometry and Statistics, University of Lübeck, 23538 Lübeck, Germany

## Aims

Genetic variants of pulmonary surface glycoproteins like Muc5a and Muc5b are known to affect mucociliary clearance, control of infections and maybe associated with pulmonary fibrosis [1–3]. We tested the hypothesis that Muc5b (rs35705950) is associated with bronchopulmonary dysplasia (BPD) in mechanically ventilated preterm infants. In addition, we aimed to identify common variants associated with the need for surfactant treatment.

## Methods

Preterm infants with a birth weight below 1500 grams born in the GNN were genotyped for rs35705950 (n=8029). Furthermore, 1272 infants were chip-genotyped (AXIOM genome wide array, GWA), and candidate polymorphisms were replicated in two additional groups of preterm infants (n=3839 and n=2830).

## Results

Frequencies for rs35705950 were in the expected range (GG 82%, GT 17%, TT 1%). The GT/TT-genotype was a strong genetic risk factor for the development of BPD in ventilated infants with sepsis (OR 5.7 95%CI 2-15; p=4.2 x 10^-5^). In GWA, we identified 10 candidate polymorphisms however, only one minor allele proved to be associated with a reduced need for surfactant treatment in preterm infants (OR 0.7, 95%CI 0.6-0.8, p=3.8 x 10^-8^ corrected for gestational age).

## Conclusion

Common polymorphisms are associated with respiratory disease in preterm infants. Our data indicate that personalized treatment stratified for genetic factors might be a useful approach for future therapy in neonates.

## Note

Wolfgang Göpel for the German Neonatal Network (GNN).
